# Molecular Epidemiology of Agents of Human Chromoblastomycosis in Brazil with the Description of Two Novel Species

**DOI:** 10.1371/journal.pntd.0005102

**Published:** 2016-11-28

**Authors:** Renata R. Gomes, Vania A. Vicente, Conceição M. P. S. de Azevedo, Claudio G. Salgado, Moises B. da Silva, Flávio Queiroz-Telles, Sirlei G. Marques, Daniel W. C. L. Santos, Tania S. de Andrade, Elizabeth H. Takagi, Katia S. Cruz, Gheniffer Fornari, Rosane C. Hahn, Maria L. Scroferneker, Rachel B. Caligine, Mauricio Ramirez-Castrillon, Daniella P. de Araújo, Daiane Heidrich, Arnaldo L. Colombo, G. S. de Hoog

**Affiliations:** 1 Microbiology, Parasitology and Pathology Post-graduation Program, Department of Basic Pathology, Federal University of Paraná, Curitiba, PR, Brazil; 2 Department of Biological Science, State University of Parana/ Campus Paranaguá, Paranaguá, PR, Brazil; 3 Department of Medicine, Federal University of Maranhão, Sao Luis, MA, Brazil; 4 Dermato-Immunology Laboratory, Institute of Biological Sciences, Federal University of Para. Marituba, PA, Brazil; 5 Clinical Hospital of the Federal University of Paraná, Curitiba, PR, Brazil; 6 University Hospital of Federal University of Maranhão, Sao Luis, MA, Brazil; 7 Cedro Laboratories Maranhão, Sao Luis, MA, Brazil; 8 Division of Infectious Diseases, Federal University of São Paulo, SP, Brazil; 9 Department of Culture Collection, Adolfo Lutz Institute, São Paulo, SP, Brazil; 10 National Institute of Amazonian Research, Manaus, Brazil; 11 Veterinary Laboratory of Molecular Biology, Faculty of Agronomy and Veterinary Medicine, Federal University of Mato Grosso, Cuiabá, MT, Brazil; 12 Department of Microbiology, ICBS, Federal University of Rio Grande do Sul, Porto Alegre, RS, Brazil; 13 Postgraduate Program in Medicine and Biomedicine, Santa Casa de Belo Horizonte Hospital, MG, Brazil; 14 Postgraduate Program in Cellular and Molecular Biology, Federal University of Rio Grande do Sul, Porto Alegre, RS, Brazil; 15 Postgraduate Program in Medicine, Federal University of Rio Grande do Sul, Porto Alegre, RS, Brazil; 16 Centraalbureau voor Schimmelcultures KNAW Fungal Biodiversity Centre, Utrecht, The Netherlands; Fundação Oswaldo Cruz, Brazil, BRAZIL

## Abstract

The human mutilating disease chromoblastomycosis is caused by melanized members of the order Chaetothyriales. To assess population diversity among 123 clinical strains of agents of the disease in Brazil we applied sequencing of the rDNA internal transcribed spacer region, and partial cell division cycle and β-tubulin genes. Strains studied were limited to three clusters divided over the single family Herpotrichiellaceae known to comprise agents of the disease. A *Fonsecaea* cluster contained the most important agents, among which *F*. *pedrosoi* was prevalent with 80% of the total set of strains, followed by 13% for *F*. *monophora*, 3% for *F*. *nubica*, and a single isolate of *F*. *pugnacius*. Additional agents, among which two novel species, were located among members of the genus *Rhinocladiella* and *Cyphellophora*, with frequencies of 3% and 1%, respectively.

## Author Summary

Chromoblastomycosis, a skin disease found among rural populations in tropical and subtropical regions, is caused by melanized fungi related to the black yeasts. The present study evaluates the species distribution among 123 clinical strains from endemic areas in Brazil based on multilocus sequence data, and describes two new agents of the disease which proved to be affiliated to *Rhinocladiella* and *Cyphellophora*.

## Introduction

Chromoblastomycosis is a chronic granulomatous infection of the skin caused by melanized fungi. It has a worldwide distribution mainly in tropical and subtropical climate zones, with a preference for humid climates with dense forestation, with *Cladophialophora carrionii* being the only species that is restricted to semi-arid areas with *Cactaceae* as main vegetation. Endemic areas are in Japan, Southeast Asia, Australia, Madagascar, as well as South and Central America [1–7]. In Brazil, the infection is observed in all states, with an estimated prevalence of 1/196 thousand inhabitants, but in some hyperendemic regions a considerably higher prevalence is noted [8]. Infection is assumed to occur through accidental inoculation of the fungus via contaminated plant debris, being favored by agricultural activities denoting an occupational nature of the disease. Chromoblastomycosis is one of the most frequent implantation mycoses found among rural populations [2, 8–11].

Clinically, the disease is characterized by pseudoepitheliomatous hyperplasia with epidermal microabscesses and dermal granulomata [12, 13]. The disease has a slow evolution, but finally may result in disfigurement of affected body sites [8]. The initial lesion is noted as a small pink papule at the site of inoculation which gradually enlarges. Development of superficial erythematous plaques with scaly or warty appearance probably takes several months or years. As a result of acanthosis these lesions may develop into large papillomatous and verrucous warts.

Species of the humid climate are particularly members of the genus *Fonsecaea*, with *F*. *pedrosoi*, *F*. *monophora* and *F*. *nubica* as prevalent agents [14–17]. Recently another species, *F*. *pugnacius* was described [18]. *Rhinocladiella aquaspersa* is an uncommon species of humid as well as of dry climates [19]. Other reported agents such as *Phialophora verrucosa* and *Exophiala dermatitidis* [17, 20] are extremely rare and mostly cause other types of infections. *Fonsecaea pedrosoi* is nearly exclusively isolated from chromoblastomycosis, while *F*. *monophora* repeatedly causes brain infection and *F*. *pugnacius* combines the two disorders by starting as chromoblastomycosis with cerebral dissemination in a single patient [18]. Species distinction of agents of the disease is clinically significant because of the differences in prognosis of the infection.

The present study evaluates the diversity of agents in endemic areas of Brazil based on multilocus sequence data, clinical aspects, direct mycological examination and culture. An enumeration of currently proven cases with molecular support in endemic areas in Brazil is provided.

## Results

**Fig 1:**
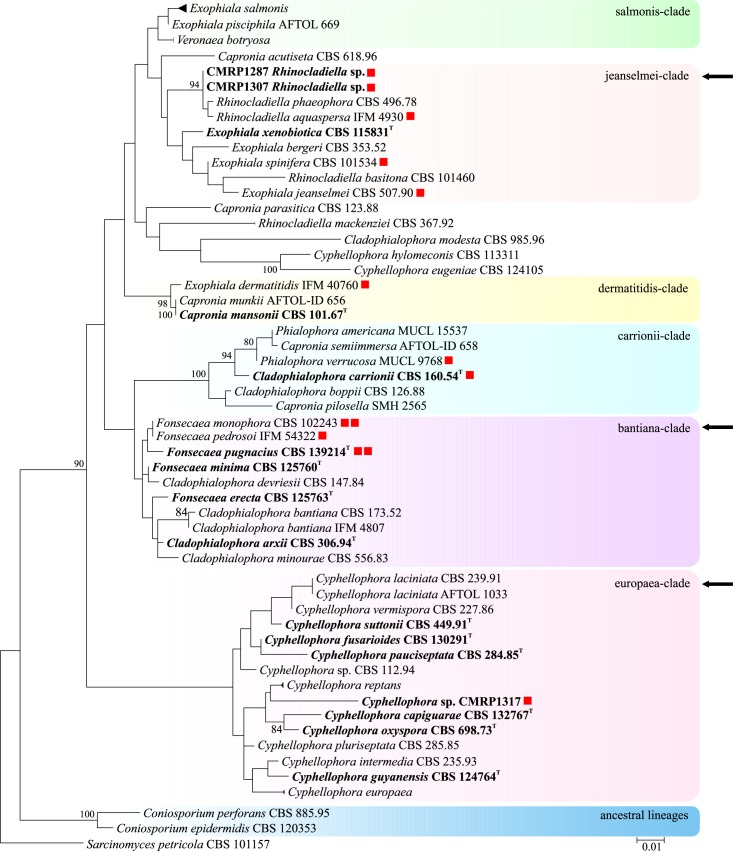
Phylogeny of a representative selection of species in Chaetothyriales, based on confidently aligned LSU sequences. Constructed with Maximum likelihood implemented in MEGA 7. Bootstrap values > 80% from 100 resampled datasets are shown with branches. Coloured boxes represent species complexes taken from de Hoog et al. [21], Feng et al. [22], and Vicente et al. [23]. Clades with species causing chromoblastomycosis analysed in this study are indicated with arrows. Type strain in bold.

A set of 123 clinical strains from cases of chromoblastomycosis from different endemic areas in Brazil was analyzed. Judging from a reference set of partial LSU rDNA sequences of members of Chaetothyriales available at CBS, agents of chromoblastomycosis were polyphyletic within the order, being dispersed in three different clades: jeanselmei-, bantiana- and europaea-clades (Fig 1, arrows).

Multilocus sequence analyses using ITS, and partial *BT2* and *CDC42* genes were performed for identification and for elucidation of species identities (S1 Fig). The 123 clinical strains clustered in different clades within the Chaetothyriales, being centred around members of the genera *Fonsecaea*, *Rhinocladiella*, and *Cyphellophora* (Table 1). The Brazilian clinical isolates used in this study all were derived from humid climate zones; members of the *Cladophialophora carrionii* group were not encountered.

**Table 1:** Strains analysed.

**Name**	**Strain number**	**Geograph/ Brazil**	**Chromoblastomycose lesions**	**ITS; BT2; CDC42; LSU**
*Fonsecaea monophora*	CMRP1290	MA, Northeast	Polymorphic lesions	KR732306; KR732312; KR732318
*Fonsecaea monophora*	CMRP1329	PR, North	Plaque	KX434631; KX583711
*Fonsecaea monophora*	CMRP1330	PR, North	Nodular	KX434632
*Fonsecaea monophora*	CMRP1359	AM, North	NI	KX434633
*Fonsecaea monophora*	CMRP1360	AM, North	NI	KX434634
*Fonsecaea monophora*	CMRP1335	MG, Southeast	NI	KX434639
*Fonsecaea monophora*	CMRP1366	PR, South	Polymorphic lesions	KX434635
*Fonsecaea monophora*	CMRP1367	RS, South	NI	KX434636
*Fonsecaea monophora*	CMRP1368	RS, South	NI	KX434637
*Fonsecaea monophora*	CMRP1369	RS, South	NI	KX434638
*Fonsecaea monophora*	CMRP1371	PR, South	NI	KX434640
*Fonsecaea monophora*	CMRP1383	PR, South	NI	KX434641
*Fonsecaea monophora*	CBS 102242	PR, South	Verrucous	EU938583; EU938549
*Fonsecaea monophora*	CBS 102243	PR, South	Scarring	EU938579; EU938542
*Fonsecaea monophora*	CBS 102246	PR, South	NI	AY366928; EU938543
*Fonsecaea monophora*	CBS 102248	PR, South	NI	AY366926; EU938550
*Fonsecaea nubica*	IAL 4004	BA, Northeast	Verrucous	KU892412
*Fonsecaea nubica*	IAL 3994	RO, North	Verrucous	KU881739
*Fonsecaea nubica*	IAL 3992	MG, Southeast	Nodular and tumoral	KU881735
*Fonsecaea nubica*	IAL 3999	SP, Southeast	Polymorphic lesions	KU8811742
*Fonsecaea pedrosoi*	CMRP1342	MA, Northeast	NI	KX434642
*Fonsecaea pedrosoi*	CMRP1271	MA, Northeast	Polymorphic lesions	KR732301; KR732307; KR732313
*Fonsecaea pedrosoi*	CMRP1272	MA, Northeast	Nodular and plaque	KR732302; KR732308; KR732314
*Fonsecaea pedrosoi*	CMRP1273	MA, Northeast	Plaque	KR732303; KR732309; KR732315
*Fonsecaea pedrosoi*	CMRP1274	MA, Northeast	Nodular and plaque	KR732304; KR732310; KR732316
*Fonsecaea pedrosoi*	CMRP1275	MA, Northeast	Plaque	KR732305; KR732311; KR732317
*Fonsecaea pedrosoi*	CMRP1276	MA, Northeast	Scarring, nodular and plaque	KX434643; -; KX583684
*Fonsecaea pedrosoi*	CMRP1277	MA, Northeast	Plaque	KX434644; KX583712; KX583685
*Fonsecaea pedrosoi*	CMRP1278	MA, Northeast	Scarring and plaque	KX434645; KX583713; KX583686
*Fonsecaea pedrosoi*	CMRP1279	MA, Northeast	Plaque	KX434646; -; KX583687
*Fonsecaea pedrosoi*	CMRP1280	MA, Northeast	Nodular and plaque	KX434647; KX583714; KX583688
*Fonsecaea pedrosoi*	CMRP1281	MA, Northeast	Nodular and plaque	KX434648
*Fonsecaea pedrosoi*	CMRP1282	MA, Northeast	Nodular and plaque	KX434649; -; KX583689
*Fonsecaea pedrosoi*	CMRP1283	MA, Northeast	Nodular and plaque	KX434650; -; KX583690
*Fonsecaea pedrosoi*	CMRP1284	MA, Northeast	Plaque	KX434651; KX583715; KX583691
*Fonsecaea pedrosoi*	CMRP1285	MA, Northeast	Plaque	KX434652; -; KX583692
*Fonsecaea pedrosoi*	CMRP1286	MA, Northeast	Scarring, nodular and plaque	KX434653; KX583716
*Fonsecaea pedrosoi*	CMRP1288	MA, Northeast	Scarring and plaque	KX434654; KX583717; KX583693
*Fonsecaea pedrosoi*	CMRP1289	MA, Northeast	Plaque	KX434655; -; KX583694
**Name**	**Strain number**	**Geograph/ Brazil**	**Chromoblastomycose lesions**	**ITS; BT2; CDC42; LSU**
*Fonsecaea pedrosoi*	CMRP1291	MA, Northeast	Plaque	KX434656; KX583718; KX583695
*Fonsecaea pedrosoi*	CMRP1292	MA, Northeast	Plaque	KX434657; KX583719; KX583696
*Fonsecaea pedrosoi*	CMRP1293	MA, Northeast	Plaque	KX434658; KX583720; KX583697
*Fonsecaea pedrosoi*	CMRP1294	MA, Northeast	Scarring and plaque	KX434659; KX583721; KX583698
*Fonsecaea pedrosoi*	CMRP1295	MA, Northeast	Scarring and plaque	KX434660; -; KX583699
*Fonsecaea pedrosoi*	CMRP1296	MA, Northeast	Scarring, nodular and plaque	KX434661; -; KX583700
*Fonsecaea pedrosoi*	CMRP1298	MA, Northeast	Plaque	KX434662; KX583722
*Fonsecaea pedrosoi*	CMRP1299	MA, Northeast	Plaque	KX434663; KX583723
*Fonsecaea pedrosoi*	CMRP1300	MA, Northeast	Scarring, nodular and plaque	KX434664
*Fonsecaea pedrosoi*	CMRP1301	MA, Northeast	Plaque	KX434665; KX583724; KX583701
*Fonsecaea pedrosoi*	CMRP1302	MA, Northeast	Scarring, nodular and plaque	KX434666; KX583725; KX583702
*Fonsecaea pedrosoi*	CMRP1303	MA, Northeast	Plaque	KX434667
*Fonsecaea pedrosoi*	CMRP1304	MA, Northeast	Polymorphic lesions	KX434668; KX583726; KX583703
*Fonsecaea pedrosoi*	CMRP1305	MA, Northeast	Nodular and plaque	KX434669; KX583727; KX583704
*Fonsecaea pedrosoi*	CMRP1306	MA, Northeast	Nodular, verrucous and plaque	KX434670; KX583728; KX583705
*Fonsecaea pedrosoi*	CMRP1308	MA, Northeast	Plaque	KX434671; KX583729; KX583706
*Fonsecaea pedrosoi*	CMRP1309	MA, Northeast	Scarring and plaque	KX434672; KX583730; KX583707
*Fonsecaea pedrosoi*	CMRP1310	MA, Northeast	Plaque	KX434673
*Fonsecaea pedrosoi*	CMRP1311	MA, Northeast	Plaque	KX434674; KX583731
*Fonsecaea pedrosoi*	CMRP1313	MA, Northeast	Plaque	KX434675; KX583732
*Fonsecaea pedrosoi*	CMRP1314	MA, Northeast	NI	KX434676; KX583733
*Fonsecaea pedrosoi*	CMRP1315	MA, Northeast	Nodular, tumoral and plaque	KX434677; KX583734
*Fonsecaea pedrosoi*	CMRP1316	MA, Northeast	Plaque	KX434678
*Fonsecaea pedrosoi*	CMRP1318	MA, Northeast	NI	KX434679
*Fonsecaea pedrosoi*	IAL 3991	BA, Northeast	Verrucous	KU881737
*Fonsecaea pedrosoi*	IAL 3997	BA, Northeast	Verrucous	KU881741
*Fonsecaea pedrosoi*	IAL 3996	BA, Northeast	Verrucous	KU881745
*Fonsecaea pedrosoi*	CMRP1331	PA, North	Nodular	KX434680
*Fonsecaea pedrosoi*	CMRP1332	PA, North	Nodular	KX434681; KX583735
*Fonsecaea pedrosoi*	CMRP1333	PA, North	Nodular	KX434682
*Fonsecaea pedrosoi*	CMRP1334	PA, North	Nodular	KX434683; KX583736
*Fonsecaea pedrosoi*	CMRP1337	PA, North	Nodular	KX434684
*Fonsecaea pedrosoi*	CMRP1338	PA, North	Nodular	KX434685; KX583737
*Fonsecaea pedrosoi*	CMRP1339	PA, North	Nodular	KX434686; KX583738
*Fonsecaea pedrosoi*	CMRP1340	PA, North	Nodular	KX434687; KX583739
*Fonsecaea pedrosoi*	CMRP1341	PA, North	Nodular	KX434688; KX583752
*Fonsecaea pedrosoi*	CMRP1344	PA, North	Nodular	KX434689; KX583740
*Fonsecaea pedrosoi*	CMRP1345	PA, North	Nodular	KX434690
*Fonsecaea pedrosoi*	CMRP1346	PA, North	Nodular	KX434691; KX583741
*Fonsecaea pedrosoi*	CMRP1347	PA, North	Nodular	KX434692
*Fonsecaea pedrosoi*	CMRP1348	PA, North	Nodular	KX434693; KX583742
*Fonsecaea pedrosoi*	CMRP1349	PA, North	Nodular	KX434694
*Fonsecaea pedrosoi*	CMRP1350	PA, North	Nodular	KX434695; KX583743
*Fonsecaea pedrosoi*	CMRP1351	PA, North	Plaque	KX434696; KX583744
*Fonsecaea pedrosoi*	CMRP1352	PA, North	Nodular	KX434697; KX583745
*Fonsecaea pedrosoi*	CMRP1353	PA, North	Nodular	KX434698; KX583746
*Fonsecaea pedrosoi*	CMRP1354	PA, North	Nodular	KX434699
*Fonsecaea pedrosoi*	CMRP1355	PA, North	Nodular	KX434700; KX583747
*Fonsecaea pedrosoi*	CMRP1356	PA, North	Nodular	KX434701
*Fonsecaea pedrosoi*	CMRP1357	PA, North	Nodular	KX434702; KX583748
**Name**	**Strain number**	**Geograph/ Brazil**	**Chromoblastomycose lesions**	**ITS; BT2; CDC42; LSU**
*Fonsecaea pedrosoi*	CMRP1361	PA, North	Nodular	KX434703
*Fonsecaea pedrosoi*	IAL 3998	RO, North	Nodular and tumoral	KU881736
*Fonsecaea pedrosoi*	IAL 4001	RO, North	Verrucous	KU881744
*Fonsecaea pedrosoi*	IAL 4005	RO, North	Polymorphic lesions	KU892413
*Fonsecaea pedrosoi*	IAL 4007	RO, North	Verrucous	KU892414
*Fonsecaea pedrosoi*	CMRP1336	MG, Southeast	NI	KX434709
*Fonsecaea pedrosoi*	CMRP1362	MS, Southeast	NI	KX434704
*Fonsecaea pedrosoi*	CMRP1363	MS, Southeast	NI	KX434705
*Fonsecaea pedrosoi*	CMRP1364	MS, Southeast	NI	KX434706
*Fonsecaea pedrosoi*	CMRP1365	MS, Southeast	NI	KX434707
*Fonsecaea pedrosoi*	IAL 3993	MG, Southeast	Nodular and tumoral	KU881738
*Fonsecaea pedrosoi*	CMRP1372	PR, South	Nodular and verrucous	KX434710
*Fonsecaea pedrosoi*	CMRP1384	PR, South	Nodular and verrucous	KX434717
*Fonsecaea pedrosoi*	CMRP1373	PR, South	Scarring	KX434711
*Fonsecaea pedrosoi*	CMRP1374	PR, South	Scarring and verrucous	KX434712
*Fonsecaea pedrosoi*	CMRP1375	PR, South	Nodular and verrucous	KX434713
*Fonsecaea pedrosoi*	CMRP1376	PR, South	Verrucous	KX434714
*Fonsecaea pedrosoi*	CBS 102245	PR, South	Scarring and verrucous	AY366918; EU938562
*Fonsecaea pedrosoi*	CBS 102247	PR, South	NI	AY366919; EU938566
*Fonsecaea pedrosoi*	CMRP1377	PR, South	Verrucous	KX434715
*Fonsecaea pedrosoi*	CMRP1378	PR, South	Plaque	KX434720
*Fonsecaea pedrosoi*	CMRP1379	PR, South	Polymorphic lesions	KX434721
*Fonsecaea pedrosoi*	CMRP1380	PR, South	Nodular and tumoral	KX434716
*Fonsecaea pedrosoi*	CMRP1381	PR, South	Nodular and tumoral	KX434718
*Fonsecaea pedrosoi*	CMRP1382	PR, South	NI	KX434719
*Fonsecaea pedrosoi*	CMRP1370	RS, South	NI	KX434708
*Fonsecaea pedrosoi*	IAL 4000	NI	Polymorphic lesions	KU881743
*Fonsecaea pedrosoi*	IAL 4006	NI	Verrucous	KU892409
*Fonsecaea pedrosoi*	IAL 4002	NI	Verrucous	KU892410
*Fonsecaea pugnacius*	CBS 139214	MA, Northeast	Plaque and disseminated to brain	KR706553; KR706547; KR706551
*Cyphellophora ludoviensis* sp. nov.	CMRP1317	MA, Northeast	Nodular and plaque	KX434722; KX583749; -; KX583708
*Rhinocladiella tropicalis* sp. nov.	CMRP1287	MA, Northeast	Polymorphic lesions	KX434723; KX583750; -; KX583709
*Rhinocladiella tropicalis*	CMRP1307	MA, Northeast	Plaque	KX434724; KX583751; -; KX583710
*Rhinocladiella tropicalis*	IMT776	SP, South	NI	KU854928

CMRP, Microbial Collections of Paraná Network- TAXon line; CBS, CBS-KNAW Fungal Biodiversity Centre, Utrecht, The Netherlands; IMT, Tropical Medicine Institute, SP, Brazil; IAL, Culture Collection of Adolfo Lutz Institute. BT2, partial beta-tubulin gene; ITS, internal transcribed spacer regions of the rDNA and intervening 5.8S nuclear ribosomal DNA (nrDNA); CDC42, partial cell division cycle gene. Brazil Sates: BA. Bahia; MA. Maranhão; MG. Minas Gerais; MS. Mato Grosso do Sul; PA. Para; RO. Rondônia; PR. Paraná; RS. Rio Grande do Sul; SP. São Paulo; NI. not informed, patients in treatment at SP.

*Fonsecaea* is nested in the ‘bantiana-clade’ (Table 1) and contains the prevalent agents of the disease. Of the studied isolates, 98 (80%) grouped as *Fonsecaea pedrosoi*, followed by 16 (13%) isolates of *F*. *monophora*, 4 (3%) isolates of *F*. *nubica* and one isolate of *F*. *pugnacius*. Isolates of the *Rhinocladiella* group and of phialophora-like species are considered as rare agents of chromoblastomycosis in the Americas. *Rhinocladiella* was nested in the ‘jeanselmei-clade’ and the phialophora-like agent clustered in the ‘europaea-clade’ (presently known as Cyphellophoraceae); two unnamed species were uncovered (Fig 1).

**Fig 2:**
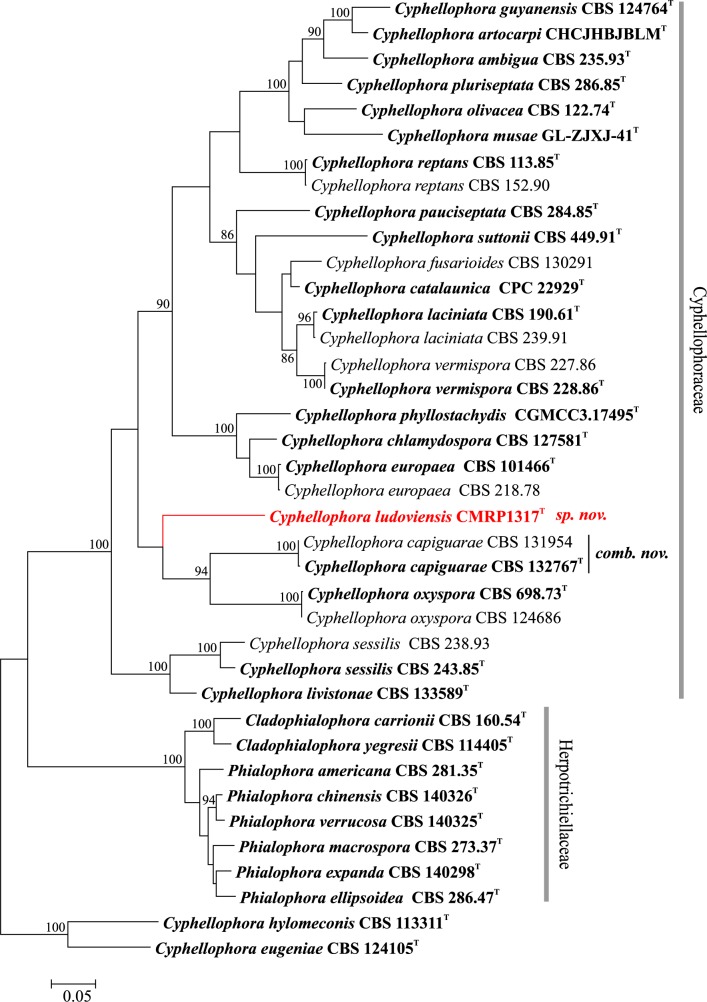
Multilocus tree of *Cyphellophora* based on ITS and partial *BT2* sequences. Constructed with maximum likelihood implemented in MEGA 7. Bootstrap values of >80% from 100 resampled data sets are shown with branches. *Cladophialophora yegresii* and *C*. *carrionii* comprised the outgroup. Novel species causing chromoblastomycosis are indicated with red branches. Type strain in bold.

**Fig 3:**
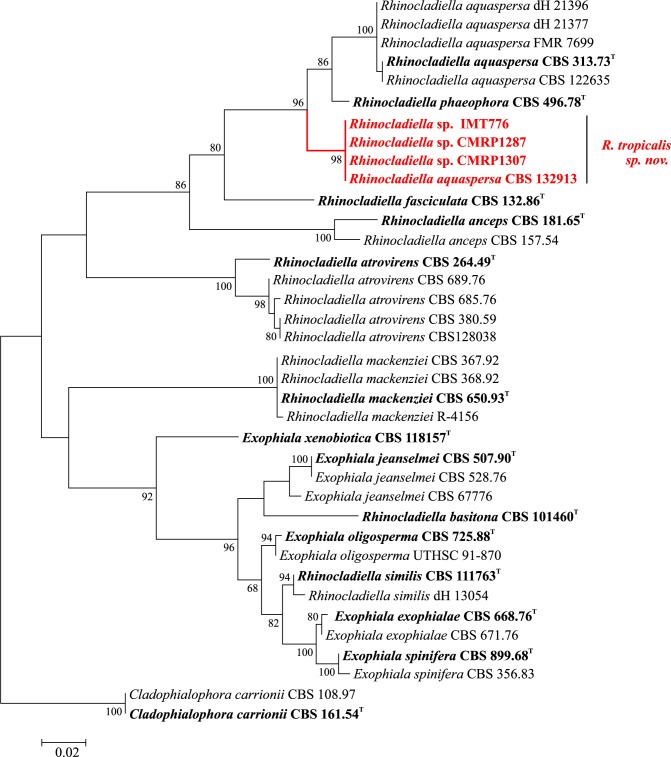
Multilocus tree of *Rhinocladiella* based on ITS and partial *BT2* sequences. Constructed with maximum likelihood implemented in MEGA 7. Bootstrap values of >80% from 100 resampled data sets are shown with branches. *Cladophialophora yegresii* and *C*. *carrionii* comprised the outgroup. Novel species causing chromoblastomycosis are indicated with red branches. Type strain in bold.

The analyzed ITS and *BT2* regions and a tree resulting from combined loci revealed identical topologies in phylogenetic analyses. The unnamed strains CMRP1287, CMRP1307, IMT776 and CMRP1317 were found to be concordantly positioned in all trees and grouped with remaining members of *Rhinocladiella* and *Cyphellophora* causing chromoblastomycosis (Figs 2 and 3). Clinical strain CMRP1317 was located at a significant distance from all reference strains of *Cyphellophora*, i.e with combined ITS and *BT2* data the distance was 23.4%. Strains CMRP1287, CMRP1307 and IMT776 were also located at significant distance from known species in *Rhinocladiella* (Table 2). Consequently the strains were judged to represent novel taxa in *Cyphellophora* and *Rhinocladiella*, respectively.

**Table 2:** Estimates of Evolutionary Divergence of *Rhinocladiella tropicalis*. Distance *R*. *aquaspersa* to *R*. *phaeophora* ITS (4.5) and *BT2* (5.6); Lengths of alignments of ITS: 548 bp and *BT2*: 349 bp. Analyses were conducted using the Kimura 2-parameter model. All ambiguous positions were removed for each sequence pair. Evolutionary analyses were conducted in MEGA 7.

Locus	*R*. *aquaspersa*	*R*. *phaeophora*	*R*. *fasciculata*	*R*. *atrovirens*	*R*. *mackenzie*	*R*. *anceps*	*R*. *basitona*	*R*. *similis*
ITS	5.1	5.4	11.4	18.6	19.7	20.4	21.7	22.0
*BT2*	9.7	10.7	----	----	----	----	34.8	36.5

**Fig 4:**
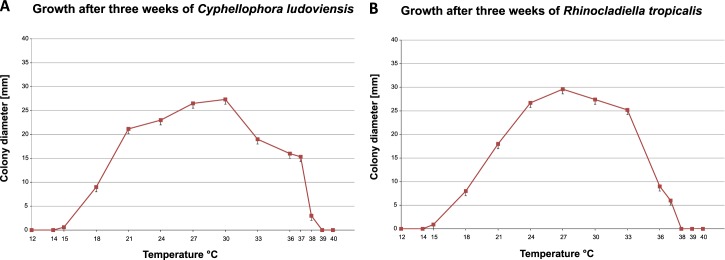
Cardinal temperatures of strains described. (A) *Cyphellophora ludoviensis* with optimal growth temperature at 30°C and maximum at 37°C. (B) *Rhinocladiella tropicalis* with optimal development at 27°C and maximum at 37°C.

We report the clinical cases caused by the novel *Cyphellophora* and *Rhinocladiella* species, which are named below as *C*. *ludoviensis* and *R*. *tropicalis*, respectively. The species showed optimal development at 30°C and 27°C, respectively, with a wide growth range between 18°C and 37°C and with residual growth at 15°C and 38°C and maximum growth temperature at 37°C; no growth was observed at 40°C (Fig 4).

**Fig 5:**
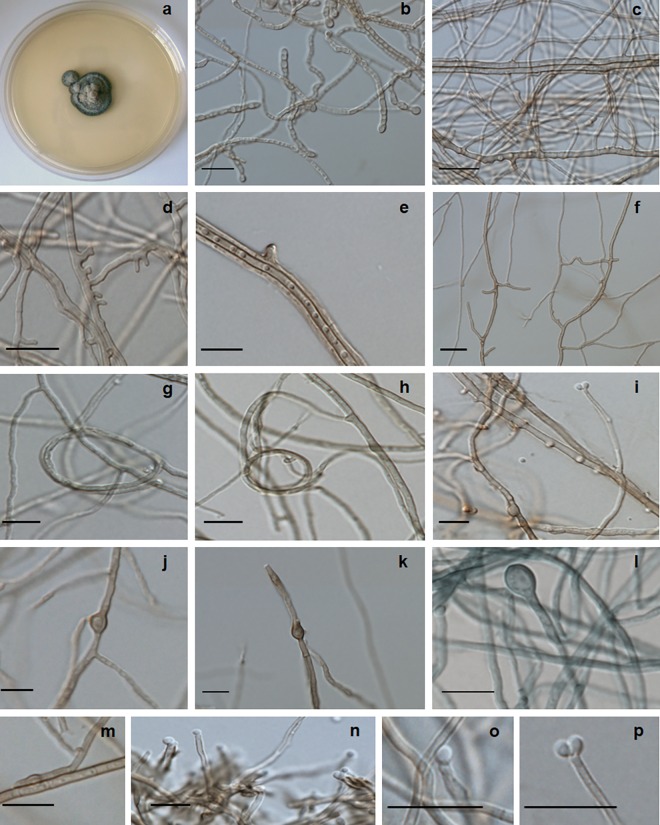
*Cyphellophora ludoviensis* microscopic morphology. (A) colonies on SGA; (B-E) hyphae with chlamydospores and lateral extensions; (F) anastomosis; (G-H) spirally twisted hyphae; (I) poorly differentiated phialide producing conidia; (J-P) chlamydospores and conidia. Scale bars 10 μm.

***Cyphellophora ludoviensis*** C.M.P.S. Azevedo, R.R. Gomes, V.A. Vicente & de Hoog, **sp. nov.** ‒ MycoBank MB 817305 (Figs 2 and 5).

Etymology: named after the city where the case was first diagnosed, San Luis in Maranhão State, Brazil.

Holotype: Maranhão State, Brazil, from skin lesion of human patient, dried holotype UPCB 85592 at Department of Botany Herbarium at Federal University of Paraná (UPCB); type strain CMRP1317 = LMICRO356 = CBM47. Additional information listed in Table 1.

Description of CMRP51317 after two weeks incubation on MEA at 28°C: Colonies moderately expanding, greyish olive to olivaceus black, with olivaceus black reverse. Hyphae pale olivaceous brown, 1.2–6 μm wide, septate every 9–25 μm, occasionally bearing scarce phialides and conidia. Phialides poorly differentiated, producing sub-spherical, hyaline conidia, 1.8−2.5 μm. Creeping hyphae producing lateral outgrowths which become septate, pale brown conidiophores 1.5−2.0 μm wide and with frequent anastomoses; chlamydospores developing intercalarily or terminally on hyphae. Chlamydospores ovoidal, brown, 4.5−6.5 × 4.5 μm, with irregularly thickened walls. Thickened terminal hyphae and spirally twisted hyphae frequently present. Teleomorph unknown. Cardinal temperatures: minimum 18°C, optimum 30°C, maximum 37°C, with residual growth at 15°C and 38°C.

**Fig 6:**
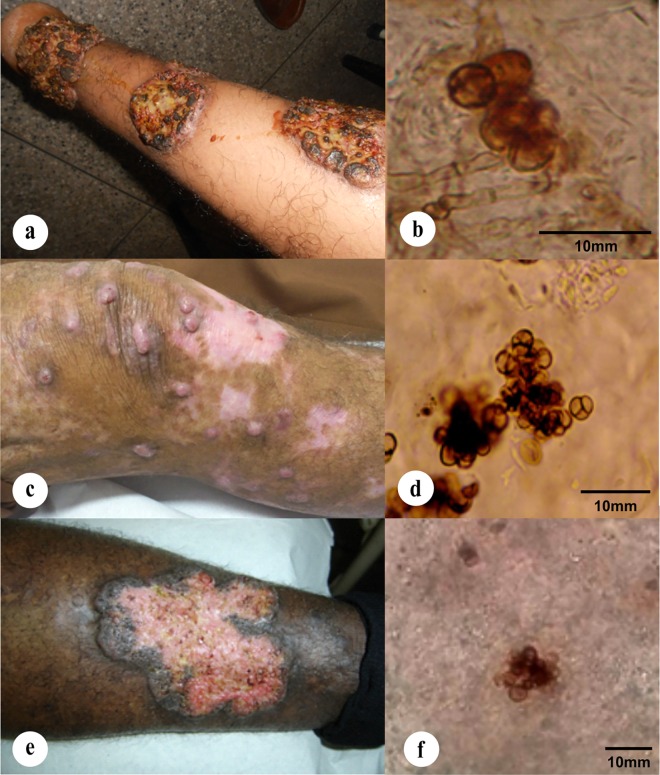
Clinical case pictures. (A, B) Nodular and verrucous lesions and muriform cells from skin tissue biopsy of arm lesion caused by *Cyphellophora ludoviensis* (CMRP1317); (C-E) polymorphic infiltrative plaque lesions caused by different strains of *Rhinocladiella tropicalis* affecting the legs, (C) with nodular and cicatricial and (E) verrucous lesions; (D-F) muriform cells from skin tissue biopsy of lesions caused by *R*. *tropicalis* strains CMRP1287 and CMRP1307, respectively.

**Notes:** The strain clustered with members of Chaetothyriales variously classified in *Cyphellophora* or *Phialophora*. Several of these have been reported from mild infections of human skin but without presence of muriform cells. *Cyphellophora ludoviensis* is the only species in the ‘europaea-clade’ (Cyphellophoraceae) where muriform cells were observed in the lesions, and is distant from remaining species causing this disease, as well as from the generic type of *Phialophora*, *P*. *verrucosa*.

**Case report:** Patient, a 57-year-old Caucasian male from Icatu, Brazil, was diagnosed in 2008 with lower limb injuries with nodular appearance and plaque. Moderate disease progressed with 3 years of evolution; muriform cells were observed in tissue (Fig 6A and 6B). Patient was treated orally with itraconazole (200 mg/day), leading to improvement of the lesions during treatment within three months. Patient abandoned treatment after 12 months and returned in 2011 with recurred lesions spreading throughout the lower limb, following a lymphatic path, with secondary infections, presenting verrucous type injuries, ulceration and crusting at the surface. The fungus was re-isolated from the recurred lesions. The patient was treated using itraconazole (200 mg/day) combined with cryosurgery.

**Fig 7:**
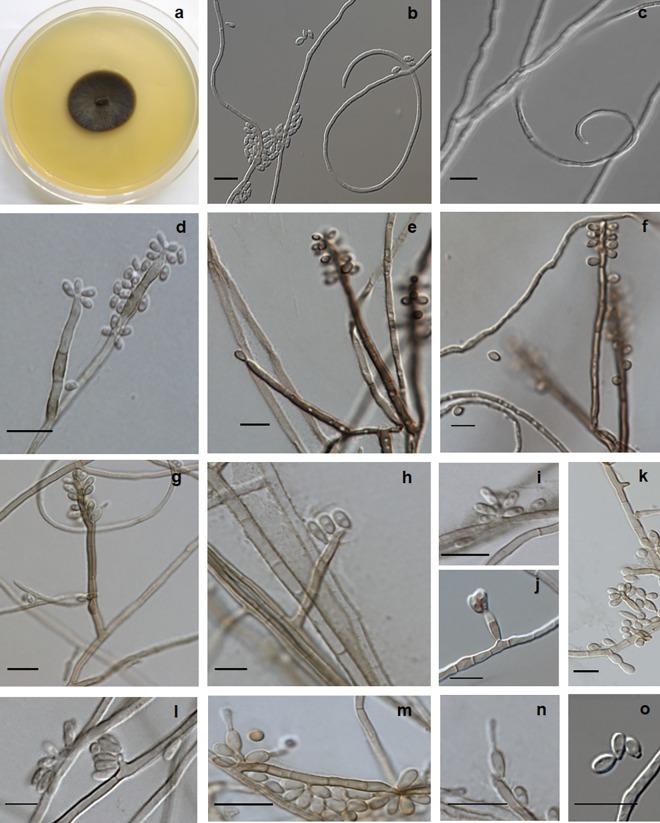
*Rhinocladiella tropicalis*, microscopic morphology. (A) Colonies on SGA; (B, C) twisted hyphae and conidia; (D-G) conidiophores with conidia produced in sympodial order; (H-O) conidial apparatus with conidia. Scale bars 10 μm.

***Rhinocladiella tropicalis*** C.M.P.S. Azevedo, R.R. Gomes, V.A. Vicente & de Hoog, **sp. nov.** ‒ MycoBank MB 817306 (Figs 3 and 7).

Etymology: Named after its occurrence in tropical South America.

Holotype: Maranhão State, Brazil, from skin lesion of human patient, dried holotype UPCB 85593 at Department of Botany Herbarium at Federal University of Paraná (UPCB); type strain CMRP1287 = LMICRO326 = CBM17. Additional material examined listed in Table 1.

Description of CMRP1287 after two weeks incubation on MEA at 28°C: Colonies growing moderate rapidly, velvety, elevated, olivaceus black with dark reverse. Mycelium partly immersed but mainly aerial, composed of branched hyphae which are pale brown, occasionally reddish brown, 1.5–2.5 μm wide, regularly septate every 7–18 μm, with integrated conidiogenous cells which are somewhat differentiated from vegetative hyphae. Conidiophores erect, straight, thick-walled, brown to dark brown, up to 19 μm high, bearing small, pigmented denticles on which conidia are produced in sympodial order. Conidiogenous cells terminal or lateral, often becoming intercalary, cylindrical in the apical part with numerous flat scars. Conidia one-celled, hyaline to pale brown, ellipsoidal to clavate, 2.5−5.0 × 1.5−2.5 μm; scars slightly prominent, approx. 0.4 μm diam, pigmented. Conidia usually 1-septate, 5−8 × 1.5−3.0 μm. Budding cells developing from single conidia may be present. Teleomorph unknown. Cardinal temperatures: minimum 18°C, optimum 27°C, maximum 37°C, with residual growth at 15°C.

**Case reports:** Patient infected by *R*. *tropicalis* CMRP1287 was a 65-year-old caucasian male from Pinheiro, Maranhão, Brazil, diagnosed in 2004 with lower limb injuries showing polymorphic lesions with plaques and nodular and cicatrized lesions. Muriform cells were observed in tissue (Fig 6C and 6D). The infection evolved during a 6-year period. Treatment was started with itraconazole (200 mg/day), irregularly until 2012; subsequently regular treatment was installed but with low response. From then onwards additional cryosurgery with liquid nitrogen was applied once a week. After 4 years of therapy still some lesions with a fibrotic aspect and with few murifom cells were observed. Patient infected by *R*. *tropicalis* CMRP1307 strain was a 78-year-old afrodescendant male, from Icatu, Maranhão, Brazil, diagnosed in 2002 with lower limb injuries showing polymorphic lesions with plaques and infiltration and muriform cells in tissue (Fig 6E and 6F). The diseased showed moderate development over a 3-year period of evolution. Patient was treated with oral itraconazole (200 mg/day), initiated in 2010. Some treatment interruptions occurred because of the distance to the patient’s living place and the low response to itraconazole, with worsening of the symptoms.

Both patients are still under treatment in 2016. The patient infected by *R*. *tropicalis* CMRP1287 is currently treated using itraconazole (200 mg/day) combined with cryosurgery and the patient infected by *R*. *tropicalis* CMRP1307 uses the antifungals itraconazole (400 mg/day) and terbinafin (250 mg twice daily) in combination.

During our studies we noticed strain IMT766, isolated in 1970 from a case of chromoblastomycosis and deposited in the Institute of Tropical Medicine, São Paulo, Brazil, and supplementary material from a patient infected by strain CBS 132913, a 63-year-old male construction worker from Venezuela with an asymptomatic and localized skin lesion of the hand with a scaly, crusted, dull-red appearance, friable with hemorrhagic dots [19].

The response to treatment has been evaluated by clinical, mycological and histopathological criteria. A complete clinical response is being accompanied assuming for cure criteria after two years follow up without recurrence, with complete healing of the lesions and disappearance of itching and local pain. The patients must also be monitored by three to four consecutive biopsies to access the mycological and histopathological criteria of cure. A mycological response will be achieved after no observation of fungal elements upon direct examination and failure to isolate the causal agent from tissue fragments.

**Notes:** With LSU rDNA the novel species clusters close to *Rhinocladiella aquaspersa* and *R*. *phaeophora* in the ‘jeanselmei clade’ in Herpotrichiellaceae (Fig 1). Members of this cluster are morphologically outstanding by stiff, erect conidiophores packed with sympodial, non-catenate conidia. With ITS (Fig 3), distances of 5.1% were noted between *R*. *tropicalis* and *R*. *aquaspersa*, and 5.4% with *R*. *phaeophora* (Table 2), underlining that a complex of sibling species is concerned.

## Discussion

In this study, we describe two novel species of black fungi causing chromoblastomycosis infections. Among a set of 123 strains from cases of this disease, representatives of three genera were recognized, i.e. *Fonsecaea*, *Rhinocladiella*, and *Cyphellophora*. No member of *Cladophialophora* was encountered, which is explained by climatic conditions, as *C*. *carrionii* is prevalent in arid environmental conditions. In Brazil *C*. *carrionii* is rarely reported [24, 25]. The prevalent species in humid tropical climates of South America remains *Fonsecaea pedrosoi*, followed by *F*. *monophora* [2], while the latter species is predominant in southern China [26]. All four species of genus *Fonsecaea* related to chromoblastomycosis were found in the Brazilian endemic area. Judging from literature data, *F*. *pedrosoi* and *F*. *nubica* seem to be pathogens that are strictly associated with chromoblastomycosis, while *F*. *monophora* and *F*. *pugnacius* show some degree of neurotropism eventually leading to dissemination to the brain and other organs [14, 27].

The genus *Cyphellophora* is characterized by phialides producing sickle-shaped, septate conidia [28]. The group clusters with some phialidic species with small, one-celled conidia. Although the generic type species of *Phialophora*, *P*. *verrucosa* clusters in the ‘carrionii-clade’ distant from *Cyphellophora*, Feng et al. [22] classified them in *Phialophora* on morphological criteria. Later Réblová et al. [29] took phylogeny as the leading principle and reclassified all species of the ‘europaea-clade’ in *Cyphellophora*, the only genus of the newly established family Cyphellophoraceae. Since the taxonomy of Chaetothyriales is in a flux with numerous species to be added, we judge establishment of categories above the species level premature. Currently, phylogenetic data do not match with any other criterion established thus far in Chaetothyriales [21], and therefore phylogenetic genera and families in this order inevitably will remain counterintuitive.

*Cyphellophora ludoviensis* is not effective to produce conidia on common mycological media; it was placed in the genus based on DNA sequence analyses. Members of the genus *Cyphellophora* colonize different habitats including, in addition to humans, plant debris, ant nests and abiotic substrates [22]. Our species is genetically close to *C*. *capiguarae*, described as living in association with ants (*Atta capiguara*), and to *C*. *oxyspora* from decaying leaves [30, 31]. Other members of *Cyphellophora* originate from mild cutaneous infections in humans, mostly from skin and nails [22]. *Cyphellophora europaea* in particular has been encountered globally as an agent of mild skin disease and onychomycosis [31] and was noted co-occurring with dermatophytes, mainly *Trichophyton rubrum* affecting the skin of diabetic patients [32]. Environmental strains of this species were found in indoor wet cells, such as bathrooms and washing machines [33, 34]. *Cyphellophora ludoviensis* is the first species in the Cyphellophoraceae that had muriform cells in tissue and showed acanthosis rather than necrosis; on the basis of these features the infection was classified as chromoblastomycosis.

The new species *Rhinocladiella tropicalis* is a cryptic species close to *R*. *phaeophora* and *R*. *aquaspersa*. *Rhinocladiella phaeophora* was known from a single strain recovered from maize field soil in Colombia [35]. It has recently been reported from a case of human chromoblastomycosis, but the sequence of this strain was not available for comparison [36]. This report nevertheless suggests that all members of the cluster consistently are able to cause chromoblastomycosis when inoculated into human skin. *Rhinocladiella aquaspersa* is a classical agent of chromoblastomycosis, nearly all cases having been reported from the American continent [19], but the majority of historical clinical cases have not been verified by sequence data. Strain CBS 132913 was originally reported as *R*. *aquaspersa* but was found to be 100% identical with *R*. *tropicalis*. The three species are phenotypically and clinically similar, but their sequence diversity interferes with molecular recognition of *R*. *aquaspersa* as a single species. According to Chen et al. [37] the term ‘species complex’ could be applied used to indicate closely related species do not seem to differ in clinically relevant parameters. The present rhinocladiella-like lineages have sufficient molecular distance to be recognized as species, but additional studies of antifungal susceptibility, clinical course, virulence and physiology are needed to verify significance of distinction of the three agents as individual species in hospital routine.

Chromoblastomycosis is clinically highly variable, with six different clinical types [8, 38]. Despite the polymorphic nature of lesions, common factor at the patient side is the absence of necrosis and often even hyper-growth of dermal tissues, which distinguishes the disease from its clinical counterpart, phaeohyphomycosis. At the fungal side, the invasive form is the muriform cell, which is likely to be the cause of the growth-promoting dermal response. As such the disease is polyphyletic within the Chaetothyriales, but has not or extremely rarely been observed outside this order. In both cases caused by these two new species here described it was encountered polymorphic lesions frequently related to clinical cases of chromoblastomycosis [8].

In the State of Maranhão, Brazil, five chaetothyrialean agents of chromoblastomycosis are endemic. A potential source of infection has been suggested [39] to be the harvest of babassu coconuts from a wild palm tree (*Orbignya phalerata*). A large part of the local rural population is involved in the collection of nuts to extract babassu oil, an important component for local and international beauty product manufacturers. Members of Chaetothyriales are indeed enriched on babassu shell fragments, which are considered a risk factor for developing chromoblastomycosis after trauma sustained at work [39, 40]. A direct link between shells and agents of the disease has however not unambiguously been established. In other Brazilian regions the number of new cases of chromoblastomycosis is decreasing [8, 41, 42]. This is thought to reflect changes in agricultural practices, the extensive use of agricultural antifungals, especially azole derivatives, and the progressive mechanization of plantations resulting in a reduction of the risk of occupational exposure [8, 43, 44].

Our results showed that *C*. *ludoviensis* and *R*. *tropicalis* had their optimal development at 30 and 27°C, respectively, the maximum growth temperature of all strains analyzed being at 37°C. The chronic nature of the infection corresponds with borderline survival of the fungus in tissue. Epidermal temperatures are usual below 37°C, allowing infection by fungi that barely support this temperature. This agrees with the clinical observation that members of the Cyphellophoraceae (‘europaea-clade’), which have their maximum growth at 36°C, cause only mild, superficial infections, having a very low degree of invasive ability and virulence [22].

According to the World Health Organization (WHO) [45] the Neglected Tropical Diseases include a series of endemic diseases that prevail in tropical or subtropical areas worldwide. The prevalence of causative microbes is linked to poverty and disadvantage. However, fungal diseases were still not included in this list, except mycetoma, another implantation mycosis [46]. Chromoblastomycosis has been reported in the literature as an overlooked disease [11]. Its global burden is comparable to or greater than that of mycetoma. Considering to its global distribution, its impact on the impoverished, and its refractoriness, it also should be considered a true neglected disease as defined by WHO.

## Materials and methods

### Strains studied

Strains analyzed comprised 123 clinical isolates (Table 1) from different cases and several endemic areas in Brazil, with viable cultures for molecular epidemiology studies and with detailed registration data deposited in medical file systems of the institutions involved in this study. All the clinical strains were deposited at Microbial Collections of Paraná Network- TAXon line at Federal University of Paraná, the register numbers, others nomenclature references and additional information were informed in the Table 1. The Holotype number was provided from Department of Botany Herbarium at Federal University of Paraná (UPCB), TAXon line collections network (http://taxonline.nerdweb.com.br/), register number at http://www.splink.org.br/.

This work was approved by the Research Ethics Committee-CEP-HUUFMA (University Hospital of the Federal University of Maranhão), according to Brazilian Resolution -Approval number: 1.276.342. All samples were anonymized. The set was supplemented with reference strains from the Centraalbureau voor Schimmelcultures (CBS/KNAW) Fungal Biodiversity Centre, Utrecht, Netherlands and the strain previously described as *R*. *aquaspersa* CBS 132913 was included in the phylogenetic analysis. Stock cultures were maintained in slants of 2% malt extract agar (MEA) and oatmeal agar (OA) at 24°C. For morphological studies, MEA and potato dextrose agar (PDA) slide cultures were prepared and mounted in aniline blue.

### Physiology

Cardinal growth temperatures were determined on 2% MEA. Plates were incubated in the dark for 3 weeks at a 3–36°C temperature range with intervals of 3°C; growth was also recorded at 14, 37, 38, 39 and 40°C. Growth rates per species were obtained by calculation of the average growth of all isolates proven to belong to that species, including the respective standard deviations. Results were plotted with temperature (°C) *versus* colony diameter (mm) as parameters. Optimum range (= average ± standard deviation) and maximum growth temperatures were determined using the type strains of each species with three replicates averages of three measurements were calculated. Different temperatures were tested, at 28°C was observed the largest number of structures to *Chyphellophora* and it was used to describe the species morphology.

### DNA extraction and amplification

DNA extraction and quality tests were performed using glass beads (Sigma G9143) according to protocols described previously [23] and when it was required, the purification of DNA was undertaken using the UltraClean™ Microbial DNA Kit (MO Bio, Carlsbad, CA, USA) according to manufacturer’s instructions. Colonies were cultivated on Sabouraud’s glucose agar (SGA).

The partial large subunit of the nuclear ribosomal RNA gene (LSU) was amplified using primers NL1 and LR5 [22] for phylogenetic assessment. Three gene regions were chosen for species delimitation: rDNA Internal Transcribed Spacer (ITS), and the partial genes cell division cycle gene (*CDC42*) and β-tubulin (*BT2*). ITS amplicons were generated with primers V9G and LS266 [47, 48] and were sequenced with primers ITS1 and ITS4. *CDC42* amplification and sequencing was generated with cdc42w and cdc42f [49] and *BT2* amplification and sequencing was generated with Bt-2a and T2 [50]. PCR was performed in a 12.5 μL volume of a reaction mixture containing 1× PCR buffer, 2.0 mM MgCl_2_, 25 μM dNTPs, 0.5 μM of each forward and reverse primers, 1 U of BioTaq DNA polymerase, and 10 ng of genomic DNA.

Amplification was performed in an ABI PRISM 2720 (Applied Biosystems, Foster City, USA) thermocycler as follows: 95°C for 4 min, followed by 35 cycles consisting of 95°C for 45s, 52°C for 30s and 72°C for 2 min, and a delay at 72°C for 7 min. Annealing temperatures were changed to 52°C, 55°C and 58°C for ITS, *CDC42* and *BT2* respectively. Amplicons were cleaned with Exonuclease I and Shrimp Alkaline Phosphatase (SAP) according to manufacturer’s instructions. Amplicons were sequenced with a BigDye Terminator Cycle Sequencing Kit v. 3.1 (Applied Biosystems, Foster City, CA, USA) according to the manufacturer’s instructions, reactions were purified with Sephadex G-50 fine (GE Healthcare Bio-Sciences, Uppsala, Sweden) and sequences were analysed on an ABI Prism 3700 DNA Sequencer (Perkin-Elmer, Norwalk, Foster City, CA, USA).

### Phylogenetic analysis

Consensus sequences of the ITS region, *BT2*, *CDC42*, and the LSU were visually inspected using MEGA v.7 software [51]. The alignment of obtained sequences was performed using the online MAFFT interface [52]. The genes ITS, *CDC42* and *BT2* were first analyzed separately and for analysis of multilocus (S1 Fig). We did the LSU analyses to assess the phylogenetic position of the species analyzed in this study. The phylogenetic analyses of the small subunit (SSU) and LSU groups previously recognized in the Herpotrichiellaceae by de Hoog et al. [21], Feng et al. [22] and Vicente et al. [23] were taken as a basis for clade delimitation. Trees were constructed with 100 bootstrap replicates using the Maximum Likelihood Implemented in Mega v. 7 software [51], with the best evolutionary model to this dataset. Conflicts were estimated using the partition homogeneity test available in PAUP* v. 4.Ob10 [53]. To elucidated and explore a more detailed the clustering unnamed species, their sequences were compared to those deposited at GenBank and the CBS-KNAW sequence data sets.

## Supporting Information

S1 Fig Multilocus tree of *Fonsecaea* and *Cladophialophora* based on confidently aligned ITS and partial *BT2* and *CDC42* sequences. Constructed with maximum likelihood implemented in MEGA 7.0. Bootstrap values of <80% from 1,000 resampled data sets are shown with branches. *Cladophialophora yegresii* (CBS 114406, CBS 114407 and CBS 114405) and *C*. *carrionii* (CBS 161.54, CBS 406.96, CBS 165.54 and CBS 108.97) comprised the outgroup. *Fonsecaea* species causing chromoblastomycosis are indicated in red. Type strain in bold.
(EPS)
