# Correction: Molecular Epidemiology of Agents of Human Chromoblastomycosis in Brazil with the Description of Two Novel Species

**DOI:** 10.1371/journal.pntd.0005315

**Published:** 2017-01-25

**Authors:** The PLOS Neglected Tropical Diseases Staff

The fifteenth author's name is spelled incorrectly. The correct name is: Rachel B. Caligiorne.

Fig 3 is incorrect. The authors have provided a corrected version here.

**Fig 3:**
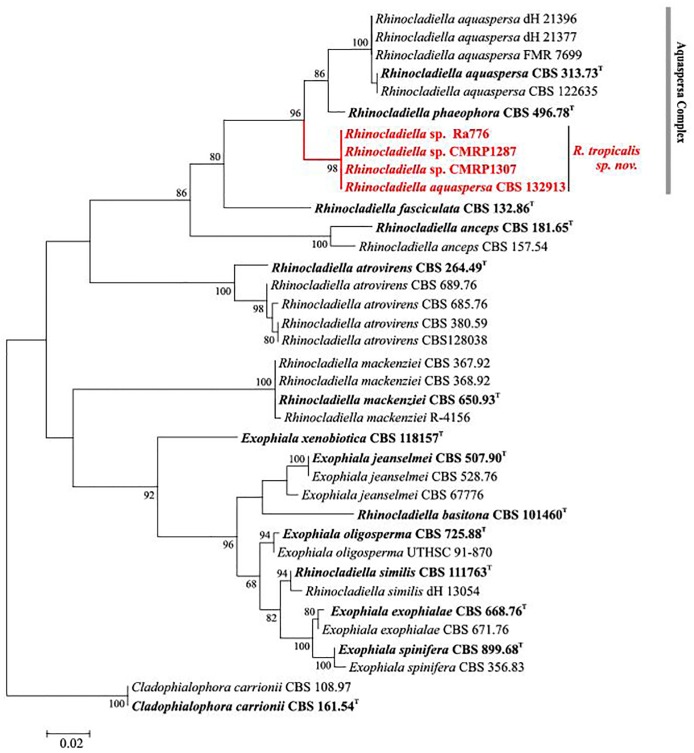
Multilocus tree of Rhinocladiella based on ITS and partial BT2 sequences. Constructed with maximum likelihood implemented in MEGA 7. Bootstrap values of >80% from 100 resampled data sets are shown with branches. *Cladophialophora yegresii* and *C. carrionii* comprised the outgroup. Novel species causing chromoblastomycosis are indicated with red branches. Type strain in bold.
